# Supplementary material for: Efficient and accurate causal inference with hidden confounders from genome-transcriptome variation data
Source: PLoS Comput Biol. 2017 Aug 18;13(8):e1005703. doi: 10.1371/journal.pcbi.1005703 (PMC5576763; doi:10.1371/journal.pcbi.1005703)
Supplement: S1 Text — (PDF) [file pcbi.1005703.s001.pdf]

Supplementary Text S1 for "Efficient and accurate causal inference with hidden confounders from genome-transcriptome variation data"

Lingfei Wang and Tom Michoel*

Division of Genetics and Genomics, The Roslin Institute, The University of Edinburgh, Easter Bush, Midlothian EH25 9RG, UK

S1 Methods

S1.1 Practical details for Bayesian inference

In practice, real PDFs are approximated with histograms. This requires a proper choice of histogram bin widths and counts. We use $\lfloor n_p^{\frac{1}{2.5}} \rfloor$ bins, capped at 100, where n_p is the total number of points used for generating the histogram. The exponent is chosen for simultaneous precision improvements from higher bin resolution and weaker fluctuation within every bin. The bin widths are also chosen as a smooth transition from uniform sample count for every bin on the 0^+ side, to uniform bin width on the positive side. (See source code for detail.)

The bin values from real data were then postprocessed to remove empty bins that are between nonempty bins, by filling them with bin values on the positive side. We then aligned the analytical null histogram by intersecting it below the postprocessed real histogram at nonzero bin values. This obtained the ratio of null hypothesis in the mixture distribution. Bayes' theorem then gave raw posterior probability $P(\mathcal{H}_{\rm alt} \mid {\rm LLR})$ at every bin center.

To enforce monotonicity of posterior distribution, we calculated the two following functions. First, starting from raw posterior probability $P(\mathcal{H}_{\rm alt} \mid {\rm LLR})$ at every bin center, set every the posterior at every bin to be no smaller than every value on its the negative side. Second, also starting from raw posterior distribution values $P(\mathcal{H}_{\rm alt} \mid {\rm LLR})$ at every bin center, set every the posterior at every bin to be no larger than every value on its the positive side. A mean is then taken between the two functions to ensure monotonicity whilst minimizing systematic bias.

We then smoothened the monotonic posterior distribution, by convolving its bin differences against a predefined normal filter, after which cumulative sum was calculated to recover the posterior distribution. A normalization was then performed to maintain the span between the previous minimum and maximum. The major purpose of smoothening is to remove duplicate values, especially those introduced during monotonicity enforcement, rather than to obtain a visually smooth function. After smoothening, we performed linear interpolation to obtain the individual post-processed posterior probabilities for each LLR.

More details can be found in the source code.

*Corresponding author. Email: Tom.Michoel@roslin.ed.ac.uk

S2 Results

S2.1 Iterative conditioning conflicts with local FDR-based probability estimation

In[1], the authors suggested that the probability of each test should be conditioned on the survival of all preceding tests, i.e. that the null distribution of each test should be estimated only on the (A, B) pairs that survive all preceding tests, although this is not implemented in Trigger package.

As an example, we applied the test combination P_2P_3 on Geuvadis dataset. After choosing an appropriate threshold probability for secondary test, as suggested in[1], we filtered only the gene pairs positive for secondary test and calculated their real and null LLR distributions of the independence test. Random permutations were applied for null distribution, with high-speed sampling from Metropolis-Hastings algorithm, whose sampling rate is exponentially increasing below secondary test's positivity threshold, and uniformly 1 above that. To balance between efficient sampling and the prevention of being trapped in local maxima, a proper exponential factor of sampling rate ($\approx 1 - n_v/n$) can be obtained from the null LLR distribution in Eq 19.

The calculation revealed that the null and real distributions form different shapes at the $\mathrm{LLR}^{(3)} \to 0^+$ side, which contradicted with the fundamental assumption of the local FDR-based probability estimation method (**Materials and methods**). On the other hand, the histograms aligned flawlessly without conditioning. We conclude that although appropriate conditioning may enhance statistical power, we are still yet to find a self-consistent approach. Since unconditioned tests have been shown self-consistent and reliable, we do not apply test conditioning in Findr.

S2.2 Analytical null distribution matches random permutations

An important feature of Findr is the novel derivation of analytical expressions for the distributions of likelihood ratio test statistics under various null distributions (**Materials and methods**). We compared the analytical null distributions to empirical null distributions obtained from random simulations. Simulated data were obtained either by permuting sample labels of the independent gene (tests 0,1,2,4), or by simulating expression levels of the gene whilst taking into account existing correlations (tests 3,5). Sufficient simulated data were then fed into the original algorithm to obtain $p(\mathrm{LLR} \mid \mathcal{H}_{\mathrm{null}})$. As demonstrated with an example in **S1 Fig**, our analytical derivation was confirmed indistinguishable with simulated distribution for miRNA hsa-miR-200b-3p's targets.

More importantly, the analytical result holds for any sample size and does not assume infinite sample sizes ($n \to \infty$). Indeed, in this asymptotic limit, the LLRs of null distributions reduce to χ^2 distributions, in agreement with Wilks's theorem[2]. For example, it is easy to confirm: $\lim_{n\to\infty} 2\,\mathrm{LLR}^{(1)} \sim \chi^2(n_v - 1)$. However, approximating LLR distributions with χ^2 leads to over-estimation of the null PDF at $\mathrm{LLR} \to 0^+$ and under-estimation at $\mathrm{LLR} \to \infty$. The tilted $p(\mathrm{LLR}|\mathcal{H}_{\mathrm{null}})$ would then cause systematic over-estimation of $P(\mathcal{H}_{\mathrm{alt}}|\mathrm{LLR})$ for all pairs. For the Geuvadis dataset with 360 samples and $n_v = 3$, an over-estimation of $\sim 1\%$ can be observed at $\mathrm{LLR} \to 0^+$. This counts an extra $\sim 1\%$ of all pairs as the alternative hypothesis, which can be of the same order as the actual percentage of true alternative hypotheses (typically at most a few percent).

S2.3 Subsampling and leaderboard performances of existing and new causal inference methods on DREAM datasets

DREAM challenge contains five datasets that have 999 samples. With numbering 1 to 5, they each contain different number of true regulations, from ~ 1000 to ~ 5000 incrementally, for the purpose of characterizing regulatory networks of different complexity. Performances on the fourth dataset are shown in the main article, and the rest here in **S2 Fig**.

We compared the performances of Findr's new test (P), Findr's correlation test (P_0), Findr's traditional causal inference test (P_T), and CIT on all 15 datasets against the published leaderboard of the DREAM5 Systems Genetics Challenge[3]. Findr's new test achieved highest AUROC and highest AUPR on all 15 datasets (**S1 Table**). It is important to note that best performers can differ on different datasets or on AUROC and AUPR. For instance, the challenge winner[4] attained best AUROC on 6/15 datasets and best AUPR on 5. When compared to other inference methods that also reported improvements[5–7], Findr demonstrates additional virtues besides the inference accuracy. Its nonparametric nature ensures robust performances across datasets without parameter tuning. Its pairwise computation scales linearly in time with the number of regulators, targets, and samples, as opposed to multivariate regression methods, providing scalability to datasets that are orders of magnitude larger.

S2.4 Findr achieves best performance on miRNA target predictions from Geuvadis dataset

We compared the performance of Findr on miRNA target prediction from the Geuvadis dataset with a suite of network inference methods that are based on gene expression data and, for some, genotype information. They include:

- All methods in the miRLAB package[8]:
  - **Correlation methods:** Pearson correlation, Spearman correlation, Kendal correlation, Distance correlation (dcov), Hoeffding's D measure (hoeffding), Randomized Dependence Coefficient (rdc), and Mutual Information (mi).
  - **Regression methods:** Lasso, and Elastic-net (elastic).
  - **Other methods:** Z-score, and Roleswitch (promise).
  - **Failed method:** Intervention calculus (ida) method failed due to excessive memory usage (greater than 16GB) and hence is excluded from comparison.
- GENIE3[9] which utilizes random forests.
- CIT[10,11] which performs multiple causal inference tests with genotype data.
- Multiple tests implemented in Findr, including: traditional (Findr-P_T), new (Findr-P), and correlation (Findr-P_0) tests.

The Trigger package[1] was not attempted because its eQTLs discovery routine exceeds both our memory and time limitations.

Their AUROCs, AUPRs, and running times are presented in **S2 Table**. The ROC and PR curves are shown in **S8 Fig**. We observed the following results:

- The correlation test topped among methods without genotype information, and in particular performs much better than Pearson and Spearman correlations. The performance gain is due to Bayesian inference, which is able to account for different gene roles such as hubs. This suggests the possibility of replacing correlation based methods with their FPR estimation counterparts in future inference of genetic regulations.

- Nonlinear methods performed worse than linear methods: mutual information v.s. Pearson correlation, and GENIE3 v.s. lasso and elastic-net. We speculate that this is due to overfitting by nonlinear methods that picked up unreal nonlinear signals from random noise and/or limited sample size, outweighing any advantage to detect genuine nonlinear effects.

- The new test P performed better than correlation test P_0. This is the first comparative study to demonstrate the effect of genotype information in the inference of gene regulatory relations.

- The traditional causal inference test performed worse than random predictions. This confirms with real data that the indirect secondary test fails to identify true but weak regulations. The independence test had negligible effect as the sample size is small (not shown in **S8 Fig**).

- Findr achieved higher AUROC and AUPR than all other methods attempted. It was also much faster than all other methods, especially CIT which also includes genotype data.

- Findr obtained a lower precision than lasso and elastic-net at small recalls. This might be explained by the fact that lasso and elastic-net are multi-variate methods which incorporate all other gene expression levels besides pairwise information, and therefore exclude indirect regulators better.

S2.5 Findr predicts transcription factor targets with accurate FDR estimates

Since CIT is much slower than Findr, with CIT we were only able to infer genetic regulations of the intersection set of the prediction and groundtruth datasets, as opposed to inferring all possible regulations with Findr.

As shown in **Fig 5**, AUPRs and AUROCs for TF target prediction with respect to known targets from siRNA silencing or TF-binding did not exhibit substantial differences, other than modest improvement over random predictions. We believe this is due to the unavoidable noise and size limitations in groundtruth data, which lead to large fluctuations in evaluation metrics and therefore could not compare methods perfectly. Furthermore, AUPR and AUROC test the entire ranked list of predictions for overlap with the groundtruth and will miss differences in enrichment for true positives between methods if they occur only among a small fraction of top-ranked predictions.

The construction of gold standard regulatory networks from TF-binding data is dependent on how TF binding sites are mapped to target genes. Here a TF regulatory interaction was assumed if

a gene had at least two binding sites for a particular TF within 10kb of its transcription start site (TSS)[12]. We repeated the analysis using the high-confidence (binding within 2.5kb) TF-target network derived from ENCODE from ChIP-sequencing data of 119 TFs in five cell types, including the lymphoblastoid cell line GM12787[13]. Fourteen TFs had a significant eQTL in the Geuvadis data. The analysis results are consistent (**Fig 5** and **S10 Fig**).

S3 Discussion

- **Existing softwares/methods not considered in this study:**

  No previous causal inference software has been able to handle the size of modern datasets. In Trigger[1], eQTL discovery and causal inference are inextricably linked, which is impractical for any sizeable dataset, especially for mammalian species; much more efficient tools for eQTL discovery have meanwhile become available[14,15]. NEO[16] is similar to CIT[11] in the tests employed, but even slower. It is able to account for multiple eQTLs, but avoids permutations using asymptotic χ^2 approximations, which result in deflated estimations for null distributions as shown in **Section S2.2**. As stated in the discussion, multi-variate network inference can be a downstream analysis based on pairwise causal inference, but evaluating such methods[17–19] fell out of the scope of this paper. Finally, note that CIT computes null distributions by permutation, separately for every gene pair, requiring months to tens of years of CPU time on a modern dataset. We were only able to include comparisons to CIT on the Geuvadis data by limiting it to the subset of gene pairs in the ground-truth tables, which correspond to 0.2% of all gene pairs for the TF target prediction case.

- **Considerations on human datasets:**

  Proving in an easily reproducible manner that the results on simulated DREAM data also hold on human data is challenging, due to the often restricted access to individual-level genotype data and the limited availability of reference databases of known interactions, especially when cell type specificity is taken into account. We performed our evaluation on the Geuvadis data, which provides transcriptome data in lymphoblastoid cells of nearly 400 individuals whose genotype data is publicly available through the 1000 Genomes project. We found that Findr predicted miRNA targets more accurately than other causal inference methods and a panel of machine learning methods that used expression data alone. Although the absolute power to predict miRNA targets may appear modest, we were primarily interested in the relative performance of various methods, and did not incorporate information about known miRNA biology, such as a preference for negative correlations or the presence of miRNA seed sequences. Moreover, since miRNAs are frequently studied in the context of diseases such as cancer, the ground-truth set of experimentally confirmed targets may represent a biased set of interactions that are not necessarily present in the lymphoblastoid cell type studied.

  To address the issue of cell type specificity, we analysed the predicted targets of 25 transcription factors for which either functional targets from siRNA silencing experiments or DNA-binding targets from ChIP-sequencing or DNase footprinting in lymphoblastoid cells were available. For both data types we found that Findr's new test achieved a 2 to 5-fold enrichment for known TF targets compared to using TF-target coexpression alone, showing that causal inference is indeed able to prioritize highly probable causal interactions among

coexpressed genes. Although Findr's new test and conditional independence based causal inference tests resulted in similar performances in this case, the estimated FDRs of the traditional method were greatly inflated, such that enrichment for known interactions was only observed at estimated local FDR >40%. This reaffirms the finding, consistently observed in all our analyses, that the conditional-independence methods are over conservative for calling causal interactions in the context of complex regulatory networks.

References

1. Lin Chen, Frank Emmert-Streib, and John Storey. Harnessing naturally randomized transcription to infer regulatory relationships among genes. *Genome Biology*, 8(10):R219, 2007.
2. Larry Wasserman. *All of statistics: a concise course in statistical inference*. Springer Science & Business Media, 2013.
3. DREAM5 Systems Genetics challenges, 2014.
4. Matthieu Vignes, Jimmy Vandel, David Allouche, Nidal Ramadan-Alban, Christine Cierco-Ayrolles, Thomas Schiex, Brigitte Mangin, and Simon De Givry. Gene regulatory network reconstruction using bayesian networks, the dantzig selector, the Lasso and their meta-analysis. *PloS one*, 6(12):e29165, 2011.
5. Marit Ackermann, Mathieu Clément-Ziza, Jacob J Michaelson, and Andreas Beyer. Teamwork: improved eQTL mapping using combinations of machine learning methods. *PloS one*, 7(7):e40916, 2012.
6. Robert J Flassig, Sandra Heise, Kai Sundmacher, and Steffen Klamt. An effective framework for reconstructing gene regulatory networks from genetical genomics data. *Bioinformatics*, 29(2):246–254, 2013.
7. Vân Anh Huynh-Thu, Louis Wehenkel, and Pierre Geurts. Gene regulatory network inference from systems genetics data using tree-based methods. *Gene Network Inference: Verification of Methods for Systems Genetics Data*, page 63, 2014.
8. Thuc Duy Le, Junpeng Zhang, Lin Liu, Huawen Liu, and Jiuyong Li. mirlab: An r based dry lab for exploring mirna-mrna regulatory relationships. *PLoS ONE*, 10(12):1–15, 12 2015.
9. Vân Anh Huynh-Thu, Alexandre Irrthum, Louis Wehenkel, and Pierre Geurts. Inferring regulatory networks from expression data using tree-based methods. *PLoS ONE*, 5(9):1–10, 09 2010.
10. Joshua Millstein, Bin Zhang, Jun Zhu, and Eric E. Schadt. Disentangling molecular relationships with a causal inference test. *BMC Genetics*, 10(1):1–15, 2009.
11. Joshua Millstein, Gary K. Chen, and Carrie V. Breton. cit: hypothesis testing software for mediation analysis in genomic applications. *Bioinformatics*, 32(15):2364–2365, 2016.
12. Darren A Cusanovich, Bryan Pavlovic, Jonathan K Pritchard, and Yoav Gilad. The functional consequences of variation in transcription factor binding. *PLoS Genetics*, 10(3):e1004226, 2014.
13. M.B. Gerstein, A. Kundaje, M. Hariharan, S.G. Landt, K.K. Yan, C. Cheng, X.J. Mu, E. Khurana, J. Rozowsky, R. Alexander, et al. Architecture of the human regulatory network derived from ENCODE data. *Nature*, 489(7414):91–100, 2012.
14. Andrey A. Shabalin. Matrix eqtl: ultra fast eqtl analysis via large matrix operations. *Bioinformatics*, 28(10):1353–1358, 2012.
15. Jianlong Qi, Hassan Foroughi Asl, Johan Bjorkegren, and Tom Michoel. krux: matrix-based non-parametric eqtl discovery. *BMC Bioinformatics*, 15(1):11, 2014.

16. Jason Aten, Tova Fuller, Aldons Lusis, and Steve Horvath. Using genetic markers to orient the edges in quantitative trait networks: The neo software. *BMC Systems Biology*, 2(1):34, 2008.
17. Bing Liu, Alberto de la Fuente, and Ina Hoeschele. Gene network inference via structural equation modeling in genetical genomics experiments. *Genetics*, 178(3):1763–1776, 2008.
18. Jun Zhu, Bin Zhang, Erin N Smith, Becky Drees, Rachel B Brem, Leonid Kruglyak, Roger E Bumgarner, and Eric E Schadt. Integrating large-scale functional genomic data to dissect the complexity of yeast regulatory networks. *Nat Genet*, 40(7):854–861, 07 2008.
19. Elias Chaibub Neto, Mark P. Keller, Alan D. Attie, and Brian S. Yandell. Causal graphical models in systems genetics: A unified framework for joint inference of causal network and genetic architecture for correlated phenotypes. *Ann. Appl. Stat.*, 4(1):320–339, 03 2010.
