# Supplementary material for: Efficient and accurate causal inference with hidden confounders from genome-transcriptome variation data
Source: PLoS Comput Biol. 2017 Aug 18;13(8):e1005703. doi: 10.1371/journal.pcbi.1005703 (PMC5576763; doi:10.1371/journal.pcbi.1005703)
Supplement: S1 Fig — Real, analytical null, and permuted null distributions are demonstrated in the figure, together with the curve of inferred posterior probability of alternative hypothesis. Permutations were randomly conducted on all potential target genes for 100 times. The alignment between analytical and permuted null distributions and the consistent incremental trend of posterior probability verify our method in deriving analytical null distributions. (PDF) [file pcbi.1005703.s002.pdf]

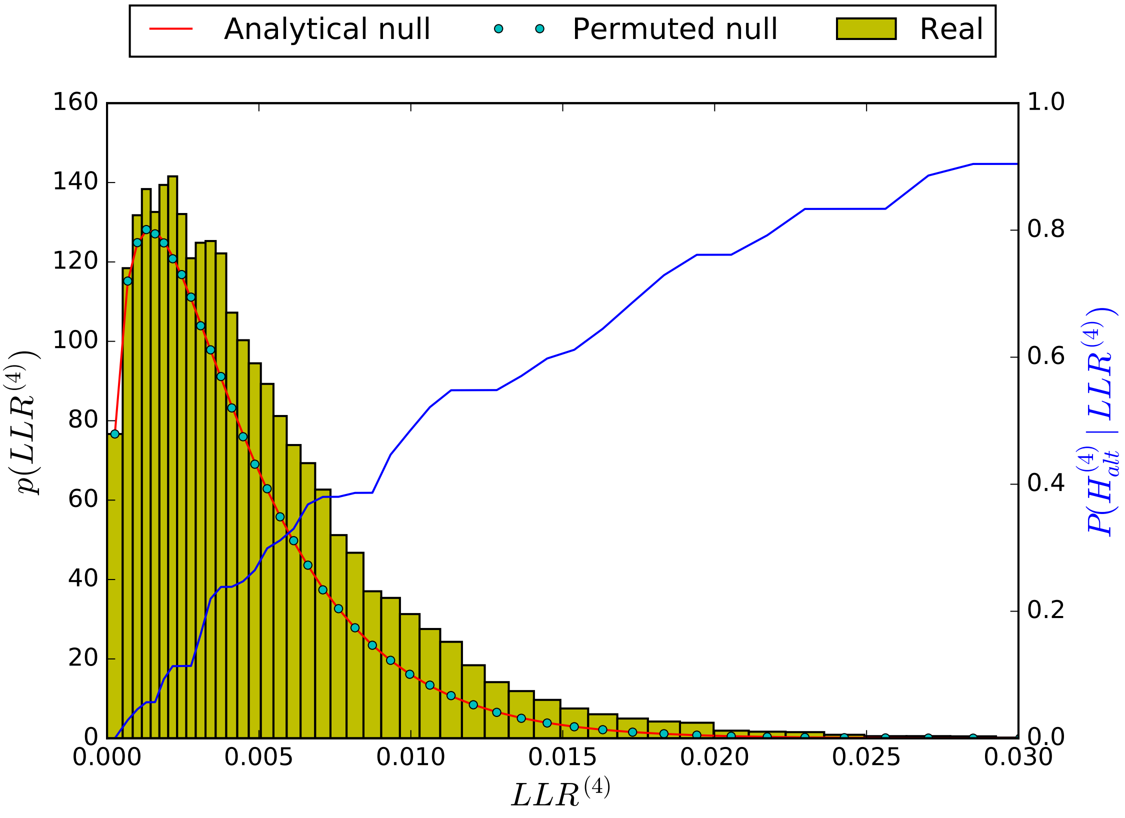

Figure S1: LLR distributions of the relevance test for hsa-miR-200b-3p on 23722 potential targets of Geuvadis dataset. Real, analytical null, and permuted null distributions are demonstrated in the figure, together with the curve of inferred posterior probability of alternative hypothesis. Permutations were randomly conducted on all potential target genes for 100 times. The alignment between analytical and permuted null distributions and the consistent incremental trend of posterior probability verify our method in deriving analytical null distributions.
