# Supplementary material for: Efficient and accurate causal inference with hidden confounders from genome-transcriptome variation data
Source: PLoS Comput Biol. 2017 Aug 18;13(8):e1005703. doi: 10.1371/journal.pcbi.1005703 (PMC5576763; doi:10.1371/journal.pcbi.1005703)
Supplement: S2 Fig — Every marker corresponds to the average AUROC or AUPR at specific sample sizes. At every sample size we performed 100 subsampling. Half widths of the lines and shades are the standard errors and standard deviations respectively, of AUROC or AUPR. Figures from top to bottom correspond to datasets 1, 2, 3, 5. For dataset 4, see Fig 2. (PDF) [file pcbi.1005703.s003.pdf]

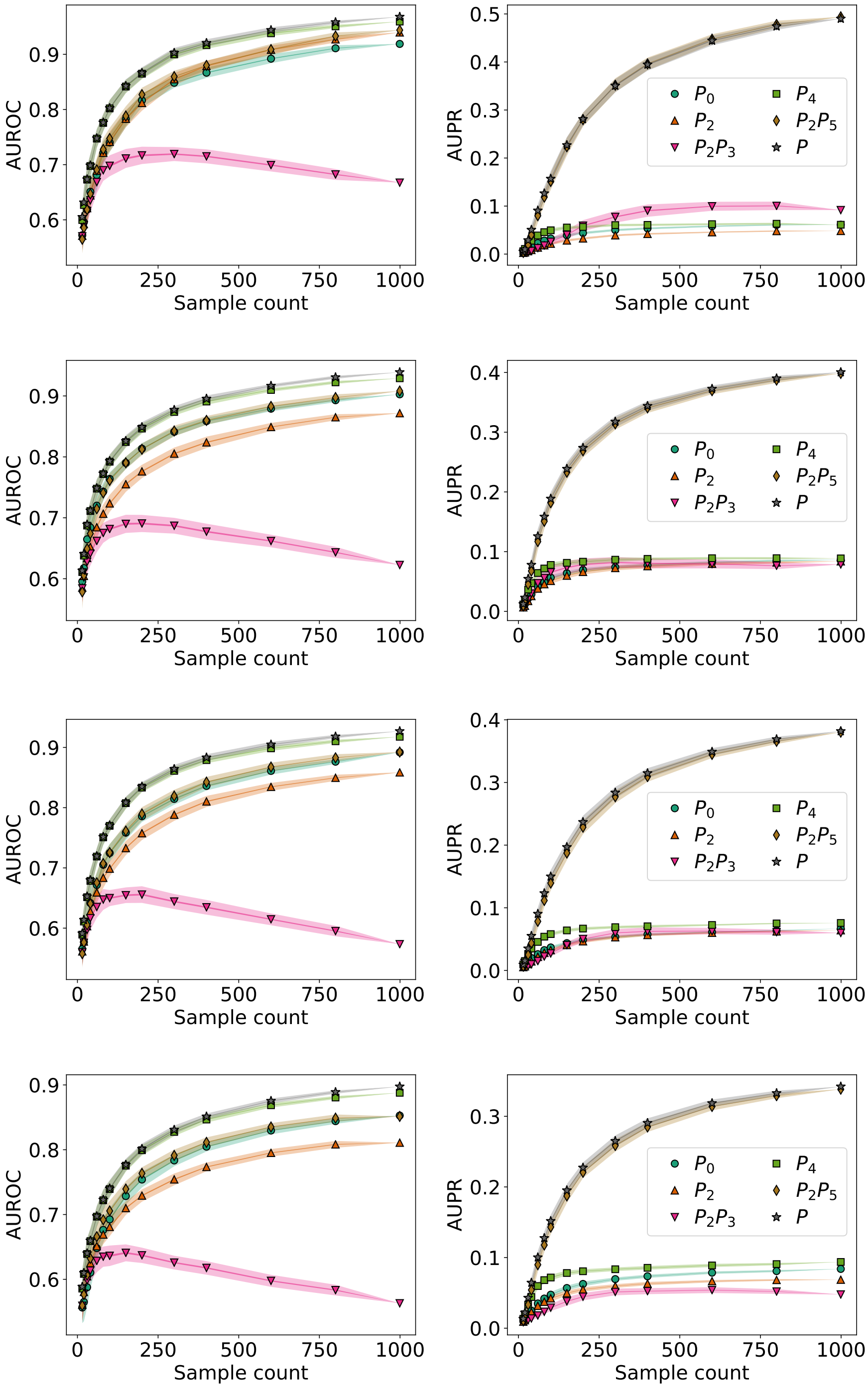

Figure S2: The mean AUROC and AUPR on subsampled data are shown for causal inference with traditional and new tests, together with the baseline correlation test. Every marker corresponds to the average AUROC or AUPR at specific sample sizes. At every sample size we performed 100 subsampling. Half widths of the lines and shades are the standard errors and standard deviations respectively, of AUROC or AUPR. Figures from top to bottom correspond to datasets 1, 2, 3, 5. For dataset 4, see **Fig 2**.
