# Supplementary material for: Efficient and accurate causal inference with hidden confounders from genome-transcriptome variation data
Source: PLoS Comput Biol. 2017 Aug 18;13(8):e1005703. doi: 10.1371/journal.pcbi.1005703 (PMC5576763; doi:10.1371/journal.pcbi.1005703)
Supplement: S3 Fig — Every marker corresponds to the AUROC or AUPR of one dataset. CIT is an R package that includes the conditional independence test, along with tests 2 and 5, while also comparing E → A → B against E → B → A. The subsampling analysis on CIT was not feasible due to its low speed. (PDF) [file pcbi.1005703.s004.pdf]

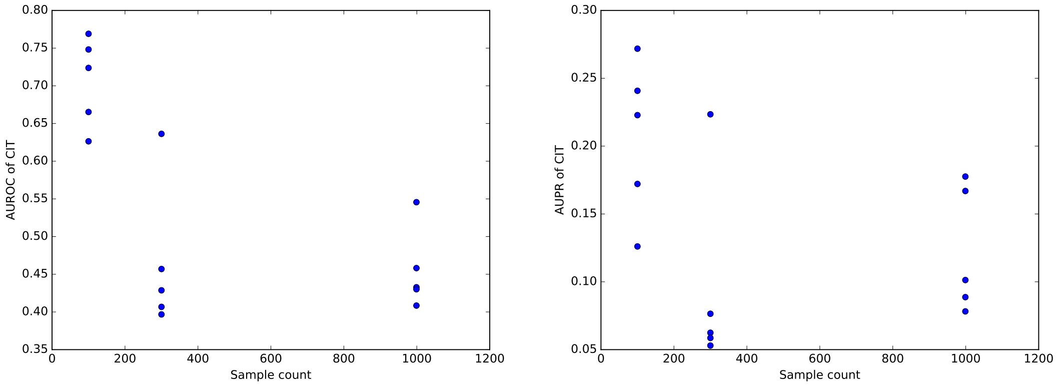

Figure S3: The AUROC and AUPR of CIT are shown for all 15 datasets of DREAM challenge. Every marker corresponds to the AUROC or AUPR of one dataset. CIT is an R package that includes the conditional independence test, along with tests 2 and 5, while also comparing $E \to A \to B$ against $E \to B \to A$. The subsampling analysis on CIT was not feasible due to its low speed.
