# Supplementary material for: Efficient and accurate causal inference with hidden confounders from genome-transcriptome variation data
Source: PLoS Comput Biol. 2017 Aug 18;13(8):e1005703. doi: 10.1371/journal.pcbi.1005703 (PMC5576763; doi:10.1371/journal.pcbi.1005703)
Supplement: S4 Fig — When A and B are both regulated by a hidden confounder C, which is independent of E (left), A becomes a collider and conditioning on A would introduce inter-dependency between E and C, which maintains E → B regulation (right). (PDF) [file pcbi.1005703.s005.pdf]

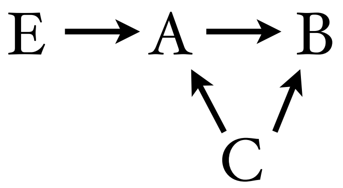

(a) With confounder before conditioning on A

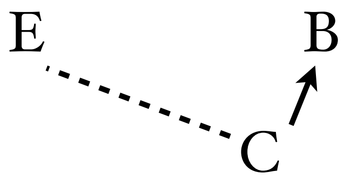

(b) With confounder after conditioning on A

Figure S4: The conditional independence test fails in the presence of hidden confounders. When A and B are both regulated by a hidden confounder C, which is independent of E (left), A becomes a collider and conditioning on A would introduce inter-dependency between E and C, which maintains $E \to B$ regulation (right).
