# Supplementary material for: Efficient and accurate causal inference with hidden confounders from genome-transcriptome variation data
Source: PLoS Comput Biol. 2017 Aug 18;13(8):e1005703. doi: 10.1371/journal.pcbi.1005703 (PMC5576763; doi:10.1371/journal.pcbi.1005703)
Supplement: S5 Fig — (PDF) [file pcbi.1005703.s006.pdf]

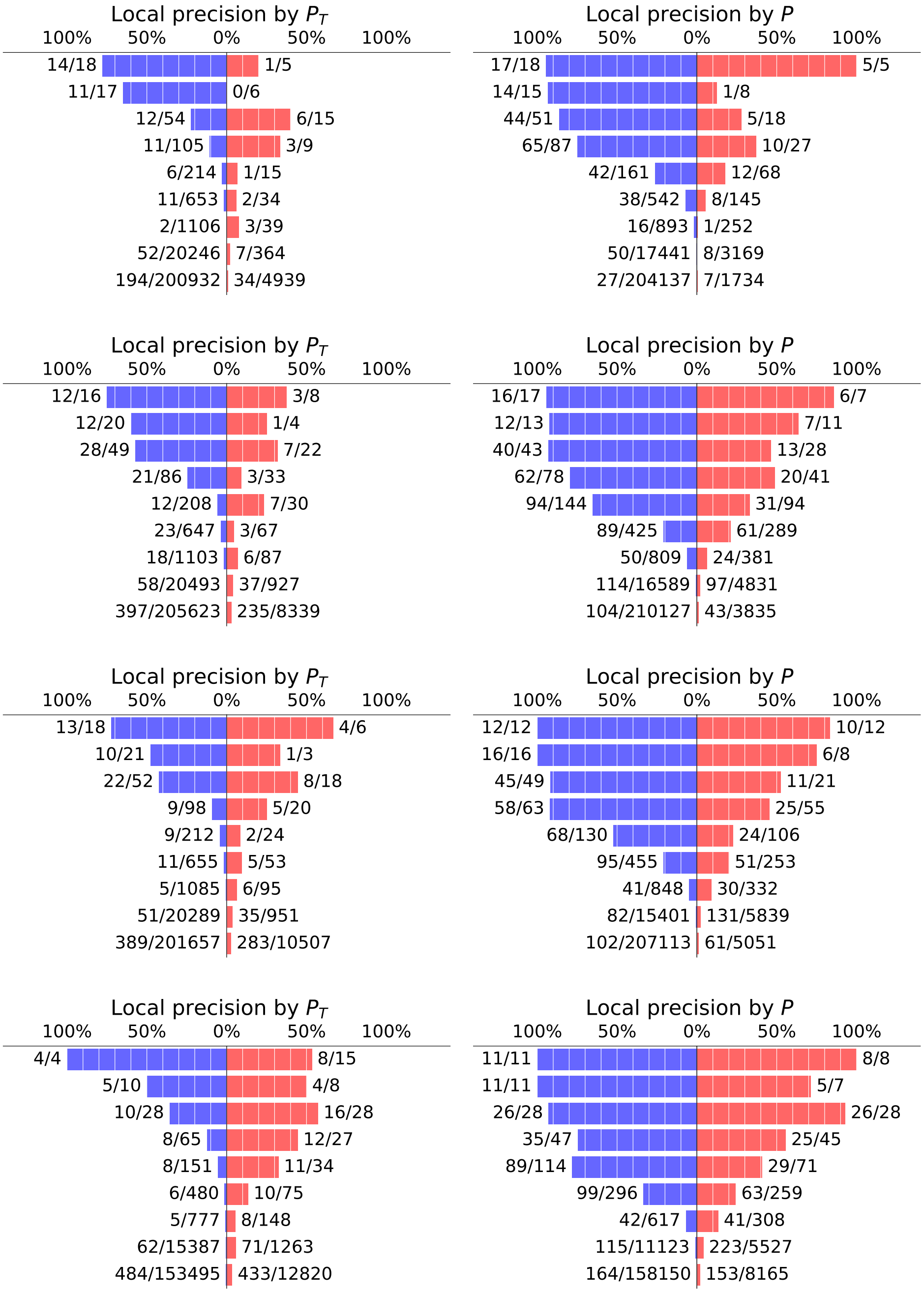

Figure S5: Local precision of top predictions for the traditional (left) and novel (right) tests for datasets (top to bottom) 1, 2, 3, and 5 of the DREAM challenge.
