# Supplementary material for: Efficient and accurate causal inference with hidden confounders from genome-transcriptome variation data
Source: PLoS Comput Biol. 2017 Aug 18;13(8):e1005703. doi: 10.1371/journal.pcbi.1005703 (PMC5576763; doi:10.1371/journal.pcbi.1005703)
Supplement: S6 Fig — The real precision was computed according to the groundtruth, whilst the estimated precision was obtained from the estimated FDR from the respective inference method (precision = 1 − FDR). Only genes with cis-eQTLs were considered as primary targets in prediction and validation. Both the novel (A, B) and the traditional (C, D) tests were evaluated. In A, C the original groundtruth table was used to validate predictions, whereas in B, D an extended groundtruth was used that also included indirect regulations at any level based on the original groundtruth. (PDF) [file pcbi.1005703.s007.pdf]

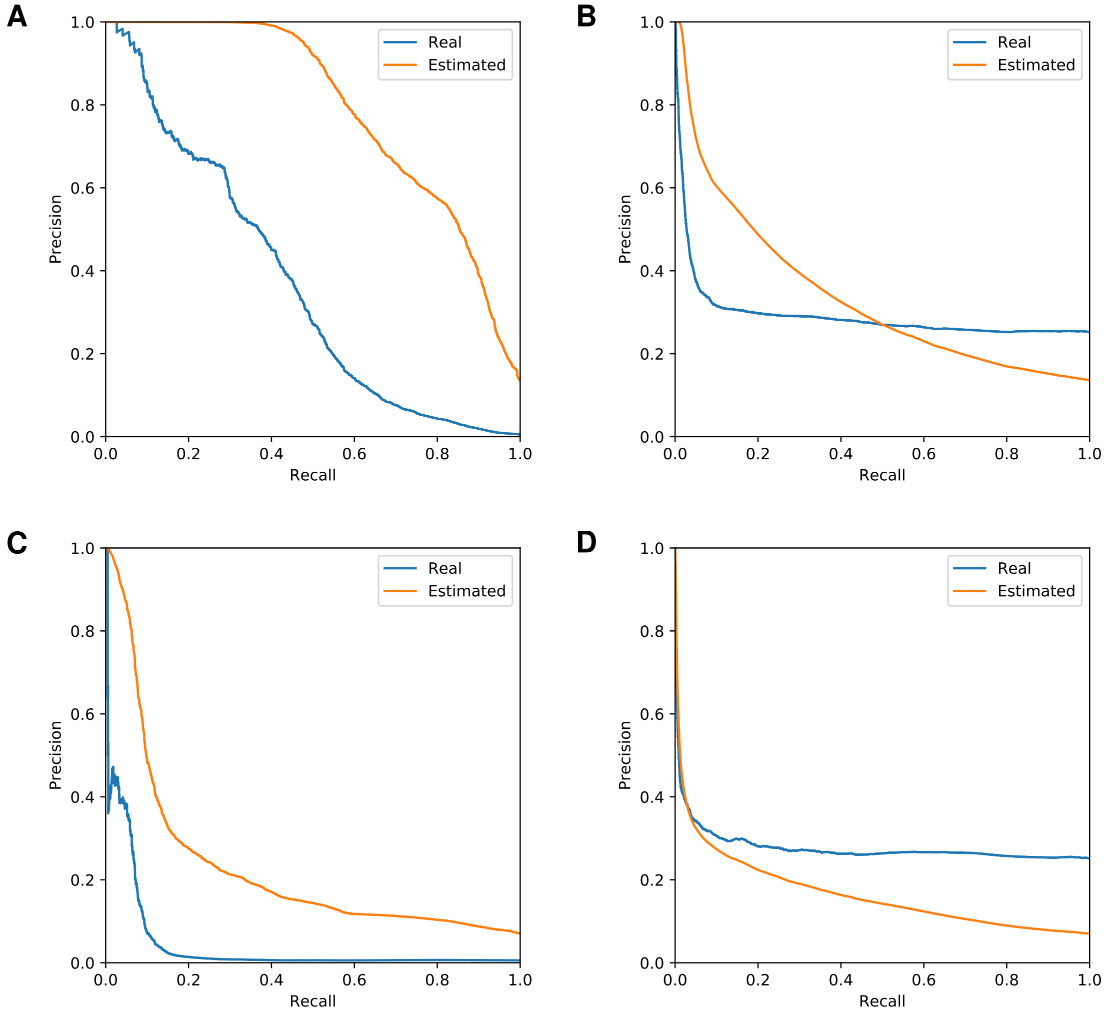

Figure S6: Estimated and real precision-recall curves for dataset 4 of the DREAM challenge. The real precision was computed according to the groundtruth, whilst the estimated precision was obtained from the estimated FDR from the respective inference method (precision $= 1-$FDR). Only genes with cis-eQTLs were considered as primary targets in prediction and validation. Both the novel (**A, B**) and the traditional (**C, D**) tests were evaluated. In **A, C** the original groundtruth table was used to validate predictions, whereas in **B, D** an extended groundtruth was used that also included indirect regulations at any level based on the original groundtruth.
