# Supplementary material for: Efficient and accurate causal inference with hidden confounders from genome-transcriptome variation data
Source: PLoS Comput Biol. 2017 Aug 18;13(8):e1005703. doi: 10.1371/journal.pcbi.1005703 (PMC5576763; doi:10.1371/journal.pcbi.1005703)
Supplement: S7 Fig — (A, B) 100 (A) or 999 (B) samples. (C, D) Minor allele frequency is 0.05 (C) or 0.3 (D). (E, F) Regarding B’s variance from A(t) → B as unit variance, B’s variance from other sources including measurement errrors is 0.2 (E) or 20 (F). Unmentioned parameters remain the same as in Fig 3. (PDF) [file pcbi.1005703.s008.pdf]

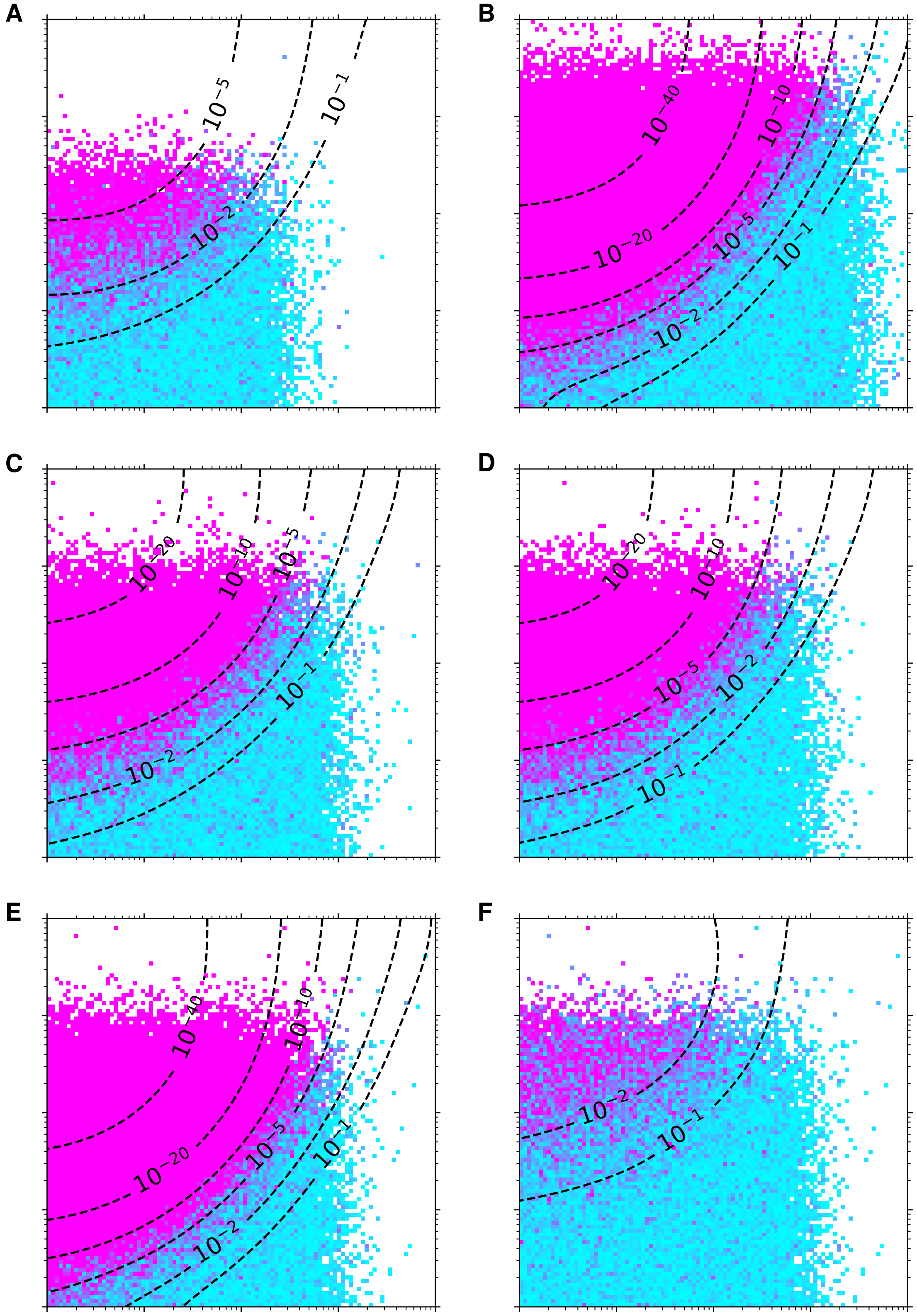

Figure S7: Null hypothesis p-values of the conditional independence test on simulated data from the ground truth model $E \to A^{(\mathrm{t})} \to B$ with $A^{(\mathrm{t})} \to A$ under parameter settings other than **Fig 3**. **(A,B)** 100 **(A)** or 999 **(B)** samples. **(C,D)** Minor allele frequency is 0.05 **(C)** or 0.3 **(D)**. **(E,F)** Regarding B's variance from $A^{(\mathrm{t})} \to B$ as unit variance, B's variance from other sources including measurement errrors is 0.2 **(E)** or 20 **(F)**. Unmentioned parameters remain the same as in **Fig 3**.
