# Supplementary material for: Efficient and accurate causal inference with hidden confounders from genome-transcriptome variation data
Source: PLoS Comput Biol. 2017 Aug 18;13(8):e1005703. doi: 10.1371/journal.pcbi.1005703 (PMC5576763; doi:10.1371/journal.pcbi.1005703)
Supplement: S8 Fig — The solid black lines correspond to expected performances from random predictions. A higher curve indicates better prediction performance. (PDF) [file pcbi.1005703.s009.pdf]

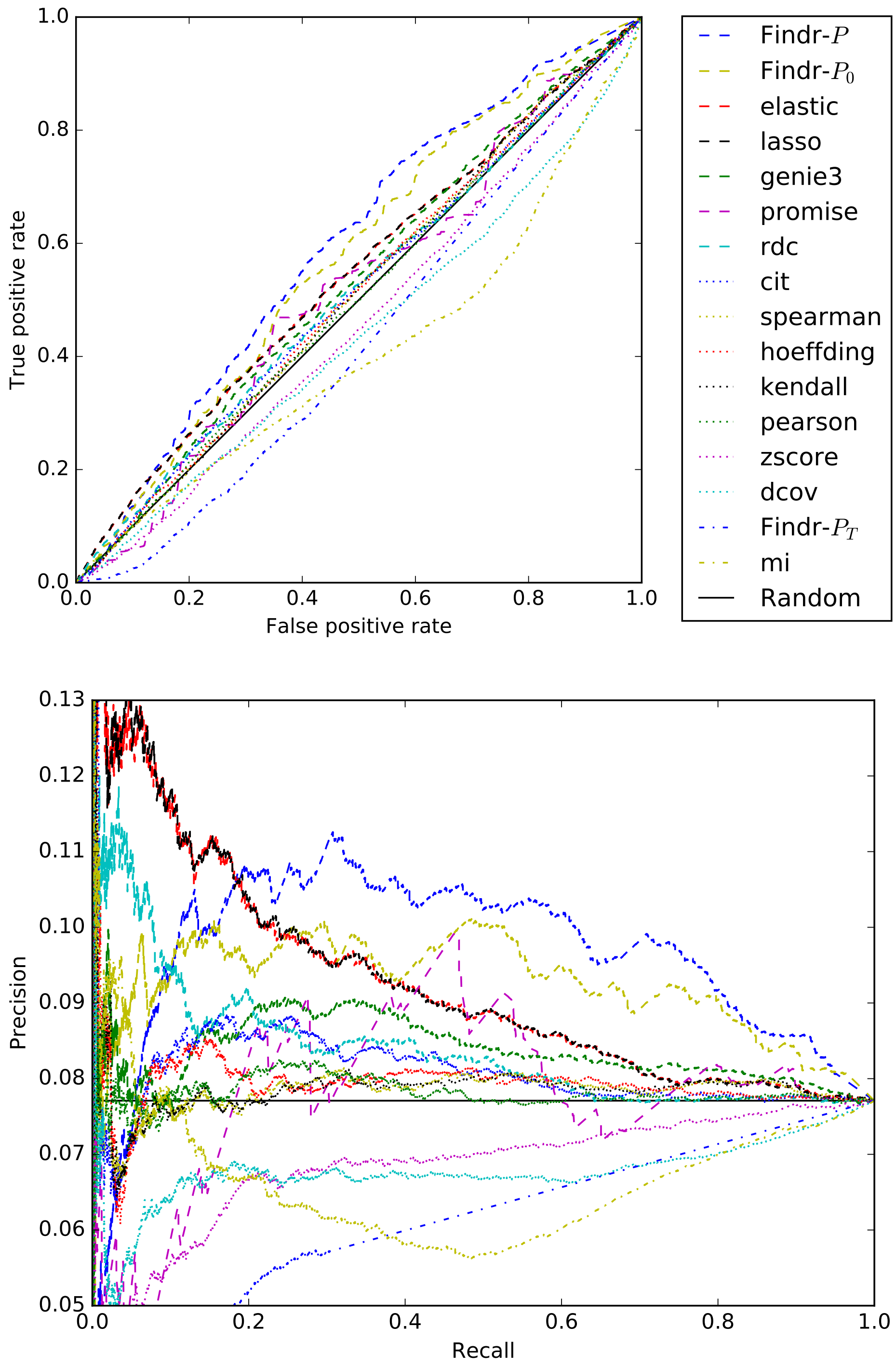

Figure S8: ROC (top) and PR (bottom) curves of miRNA target predictions were compared for Findr's traditional, new, and correlation tests, GENIE3, CIT, and 11 methods in miRLAB, based on Geuvadis data. The solid black lines correspond to expected performances from random predictions. A higher curve indicates better prediction performance.
