# Supplementary material for: Efficient and accurate causal inference with hidden confounders from genome-transcriptome variation data
Source: PLoS Comput Biol. 2017 Aug 18;13(8):e1005703. doi: 10.1371/journal.pcbi.1005703 (PMC5576763; doi:10.1371/journal.pcbi.1005703)
Supplement: S9 Fig — AUROC and AUPR metrics are measured for three inference tasks. MiRNA compares miRNA target predictions based on Geuvadis miRNA and mRNA expression levels against groundtruths from miRLAB. SiRNA and TF-binding compares gene-gene interaction predictions based on Geuvadis gene expression levels against groundtruths from siRNA silencing and TF-binding measurements respectively. ENCODE compares the same gene-gene interaction predictions against TF-binding networks derived from ENCODE data. Dashed lines indicate expected performances from random predictions. (PDF) [file pcbi.1005703.s010.pdf]

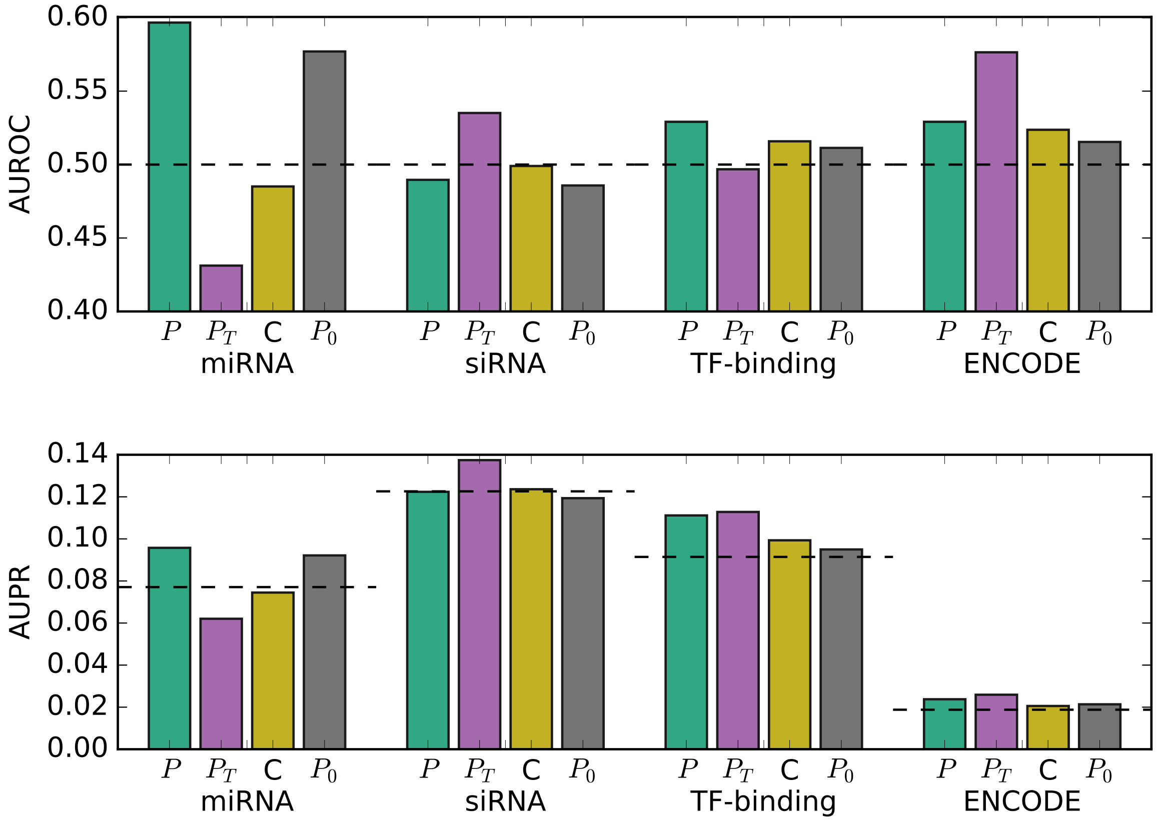

Figure S9: Three methods of causal inference were evaluated and compared against the baseline correlation test method (P_0): Findr's new test (P), traditional causal inference test in Findr (P_T), and CIT (C). AUROC and AUPR metrics are measured for three inference tasks. MiRNA compares miRNA target predictions based on Geuvadis miRNA and mRNA expression levels against groundtruths from miRLAB. SiRNA and TF-binding compares gene-gene interaction predictions based on Geuvadis gene expression levels against groundtruths from siRNA silencing and TF-binding measurements respectively. ENCODE compares the same gene-gene interaction predictions against TF-binding networks derived from ENCODE data. Dashed lines indicate expected performances from random predictions.
