# Supplementary material for: Efficient and accurate causal inference with hidden confounders from genome-transcriptome variation data
Source: PLoS Comput Biol. 2017 Aug 18;13(8):e1005703. doi: 10.1371/journal.pcbi.1005703 (PMC5576763; doi:10.1371/journal.pcbi.1005703)
Supplement: S10 Fig — The number above each bar indicates the number of positive predictions at the corresponding threshold. The dashed line is precision from random predictions. (PDF) [file pcbi.1005703.s011.pdf]

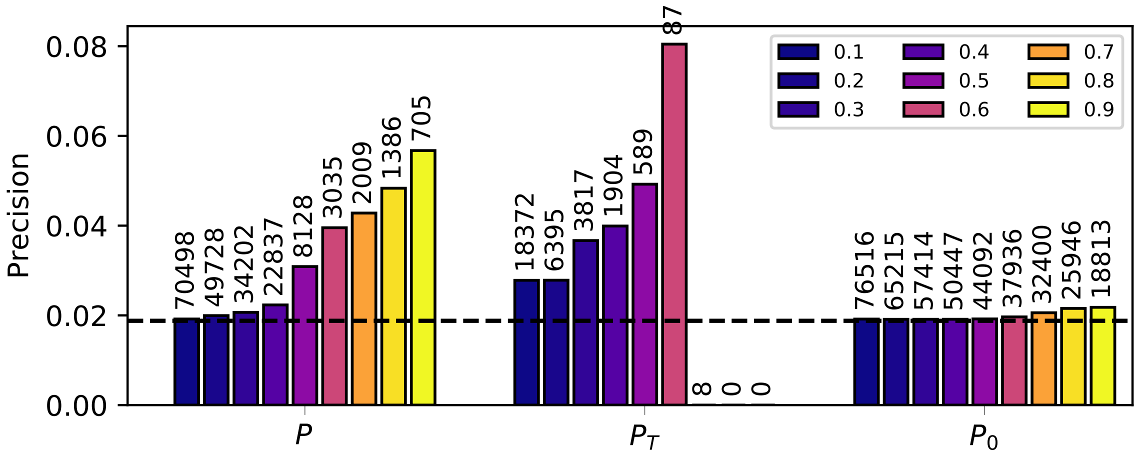

Figure S10: Inference precision at estimated precision cutoffs 0.1 to 0.9 with respect to groundtruth network derived from TF binding of 14 TFs from ENCODE data. The number above each bar indicates the number of positive predictions at the corresponding threshold. The dashed line is precision from random predictions.
