# Supplementary material for: Efficient and accurate causal inference with hidden confounders from genome-transcriptome variation data
Source: PLoS Comput Biol. 2017 Aug 18;13(8):e1005703. doi: 10.1371/journal.pcbi.1005703 (PMC5576763; doi:10.1371/journal.pcbi.1005703)
Supplement: S1 Table — All cis- and trans-genes are included. DREAM challenge constrained the maximum number of submitted regulations by 100,000, which were also applied in our evaluation. Findr’s new test consistently obtained higher AUROC and AUPR than all other methods, including the leaders of DREAM challenge. (PDF) [file pcbi.1005703.s012.pdf]

Table S1: Predictions from Findr's new (P), traditional (P_T), and correlation (P_0) tests, and CIT were compared against DREAM challenge leaders on AUROC and AUPR for all 15 DREAM datasets. All cis- and trans-genes are included. DREAM challenge constrained the maximum number of submitted regulations by 100,000, which were also applied in our evaluation. Findr's new test consistently obtained higher AUROC and AUPR than all other methods, including the leaders of DREAM challenge.

Sample count	100 samples					300 samples					999 samples				
Test id	1	2	3	4	5	1	2	3	4	5	1	2	3	4	5
P AUROC	**0.772**	**0.750**	**0.737**	**0.736**	**0.719**	**0.882**	**0.842**	**0.839**	**0.825**	**0.797**	**0.941**	**0.899**	**0.882**	**0.867**	**0.848**
P_T AUROC	0.617	0.609	0.597	0.574	0.572	0.647	0.594	0.581	0.592	0.573	0.616	0.611	0.570	0.616	0.565
P_0 AUROC	0.709	0.706	0.706	0.699	0.700	0.843	0.798	0.803	0.792	0.766	0.905	0.870	0.850	0.837	0.813
CIT AUROC	0.585	0.582	0.571	0.548	0.569	0.630	0.586	0.575	0.574	0.566	0.614	0.640	0.594	0.614	0.577
Leader AUROC	0.754	0.718	0.699	0.694	0.688	0.861	0.793	0.799	0.769	0.757	0.933	0.885	0.845	0.828	0.813
P AUPR	**0.222**	**0.183**	**0.172**	**0.161**	**0.155**	**0.421**	**0.326**	**0.279**	**0.264**	**0.258**	**0.547**	**0.368**	**0.366**	**0.342**	**0.333**
P_T AUPR	0.044	0.048	0.042	0.023	0.041	0.109	0.054	0.051	0.054	0.047	0.070	0.068	0.042	0.070	0.049
P_0 AUPR	0.051	0.049	0.040	0.051	0.055	0.084	0.053	0.063	0.078	0.057	0.093	0.072	0.070	0.076	0.077
CIT AUPR	0.075	0.066	0.060	0.031	0.050	0.162	0.075	0.074	0.080	0.067	0.168	0.149	0.098	0.168	0.096
Leader AUPR	0.103	0.072	0.067	0.068	0.067	0.309	0.243	0.191	0.182	0.191	0.358	0.258	0.195	0.183	0.178
