# Supplementary material for: Efficient and accurate causal inference with hidden confounders from genome-transcriptome variation data
Source: PLoS Comput Biol. 2017 Aug 18;13(8):e1005703. doi: 10.1371/journal.pcbi.1005703 (PMC5576763; doi:10.1371/journal.pcbi.1005703)
Supplement: S2 Table — Higher AUROC and AUPR values signify stronger predictive power. Program running times have units in seconds (s), minutes (m), hours (h), or days (d). Findr outperformed other methods in statistical power and speed, with or without genotype information. (PDF) [file pcbi.1005703.s013.pdf]

Table S2: AUROCs and AUPRs of miRNA target predictions were compared for Findr's traditional, new, and correlation tests, GENIE3, CIT, and 11 methods in miRLAB, based on Geuvadis data. Higher AUROC and AUPR values signify stronger predictive power. Program running times have units in seconds (s), minutes (m), hours (h), or days (d). Findr outperformed other methods in statistical power and speed, with or without genotype information.

	Findr-P	Findr-P_0	elastic	lasso	genie3	promise
AUROC	0.60	0.58	0.55	0.54	0.53	0.52
AUPR	0.096	0.092	0.092	0.092	0.083	0.078
Time	0.88s	0.30s	4.53m	4.47m	12.1h	2.37m
	rdc	cit	spearman	hoeffding	kendall	pearson
AUROC	0.52	0.51	0.51	0.51	0.51	0.50
AUPR	0.083	0.080	0.078	0.079	0.078	0.078
Time	50.3m	7.5d	2.37m	16.3m	41.9m	2.27m
	random	zscore	dcov	Findr-P_T	mi	
AUROC	0.50	0.46	0.44	0.43	0.41	
AUPR	0.077	0.068	0.068	0.062	0.068	
Time	-	2.90m	4.42h	0.84s	23.1m	
