# Supplementary material for: Efficient and accurate causal inference with hidden confounders from genome-transcriptome variation data
Source: PLoS Comput Biol. 2017 Aug 18;13(8):e1005703. doi: 10.1371/journal.pcbi.1005703 (PMC5576763; doi:10.1371/journal.pcbi.1005703)
Supplement: S3 Table — The three gold standards could not agree on method. (PDF) [file pcbi.1005703.s014.pdf]

Table S3: AUROCs and AUPRs of gene target predictions were compared for a selected subset of methods in **S2 Table**, also based on Geuvadis data. The three gold standards could not agree on method accuracy.

Gold standard	siRNA		TF-binding		ENCODE	
Metric	AUROC	AUPR	AUROC	AUPR	AUROC	AUPR
Random	0.500	0.123	0.500	0.0914	0.500	0.0188
Findr-P	0.490	0.122	0.529	0.1112	0.529	0.0238
Findr-P_T	0.535	0.137	0.497	0.1128	0.576	0.0259
Findr-P_0	0.486	0.119	0.510	0.0947	0.516	0.0214
CIT	0.499	0.124	0.515	0.0993	0.524	0.0206
elastic	0.501	0.122	0.508	0.0941	0.504	0.0194
lasso	0.500	0.122	0.508	0.0943	0.504	0.0193
genie3	0.499	0.124	0.492	0.0902	0.517	0.0206
spearman	0.496	0.122	0.510	0.0936	0.520	0.0211
pearson	0.494	0.122	0.516	0.0958	0.514	0.0209
zscore	0.497	0.121	0.527	0.1007	0.508	0.0194
mi	0.500	0.122	0.483	0.0863	0.491	0.0186
